# Connected Hearing Devices and Audiologists: The User-Centered Development of Digital Service Innovations

**DOI:** 10.3389/fdgth.2021.739370

**Published:** 2021-09-20

**Authors:** Marie Luengen, Christopher Garrelfs, Kamil Adiloǧlu, Melanie Krueger, Benjamin Cauchi, Uwe Markert, Marei Typlt, Martin Kinkel, Carsten Schultz

**Affiliations:** ^1^Department for Technology Management, Kiel University, Kiel, Germany; ^2^HörTech gGmbH, Oldenburg, Germany; ^3^OFFIS e.V., Institute for Information Technology, Oldenburg, Germany; ^4^Audifon GmbH & Co. KG, Kölleda, Germany; ^5^KIND GmbH & Co. KG, Burgwedel, Germany

**Keywords:** service innovation, user-centered design, integrated service platform, remote fitting, machine learning

## Abstract

Today, medical technology manufacturers enter the service market through the development of digital service innovations. In the field of audiology, these developments increasingly shift the service capacities from audiologists to manufacturers and technical systems. However, the technology-driven developments of manufacturers lack acceptance of hearing device users and undermine the important role of audiologists within the service provision. By following a user-centered design approach in order to deal with the technological and social challenges of disruptive services, we aim to develop service innovations on an integrated service platform in the field of tele-audiology. To ensure the acceptance of technology-driven service innovations among hearing device users and audiologists, we systematically integrated these actors in a participatory innovation process. With qualitative and quantitative data we identified several requirements and preferences for different service innovations in the field of tele-audiology. According to the preferences of the different actors, we proposed a service platform approach based on a connected hearing device in three pillars of application: 1) one-to-one (1:1) service innovations based on a remote fitting concept directly improve the availability of services offered by audiologists without being physically present. Based on this, 2) one-to-many (1:N) service innovations allow the use of the connected hearing device as an indirect data source for training a machine learning algorithm that empowers users through the automation of service processes. A centralized server system collects the data and performs the training of this algorithm. The optimized algorithm is provided to the connected hearing devices to perform automatic acoustic scene classification. This in turn allows optimization of the hearing devices within each acoustic scene. After the user-centered development of the different service innovations which are designed to converge on an integrated service platform, we experimentally evaluated the functionality and applicability of the system as well as the associated role models between the technical system, the hearing device users and audiologists. As a future outlook, we show potentials to use the connected hearing device for 3) cross-industry (N:M) service innovations in contexts outside the healthcare domain and give practical implications for the market launch of successful service innovations in the field of tele-audiology.

## Introduction

### Digital Service Innovations in Audiology

According to the World Report on Hearing (1), hearing difficulties are among the most common diseases worldwide. Unaddressed hearing loss is the third largest cause of years lived with disability globally. Over 1.5 billion people currently experience some degree of hearing loss, with over 400 million people living with disabling hearing loss. Only one out of five people suffering from hearing difficulties is using any type of supporting device today. Although hearing difficulties affect all ages, the prevalence increases for elderly people. Due to an aging population, the number of people with at least some degree of hearing difficulties will continuously grow in the future. Through the use of new digital technologies, increasing attention has been paid to the development of service innovations in healthcare (2, 3). Artificial intelligence (AI), big data analysis, Internet of Things (IoT) and the smartification of products into product-service-systems enable healthcare organizations to find new ways of value creation (4).

In contrast to traditional services, digital services are characterized by high levels of re-programmability, homogeneity and self-reference (5, 6). Re-programmability enables the subsequent adaptation and extension of an already implemented service solution (7). Homogeneity includes the possibility to store and transmit digital information and processes which allow for more scalability, broader user targeting, and faster strategic actions (6). Finally, digital services reinforce the diffusion and development of other digital services and technologies which lead to lower entry barriers for future service developments and an even higher diffusion of such service offerings. The field of audiology is predestined for service innovations aiming at increasing the rate of candidates using hearing devices [HDs, e.g., hearing aids (HAs), cochlear implants (CIs)] and improving the quality of current service processes. As such, service organizations within the field of audiology can substantially benefit from service innovations. Especially in the field of tele-audiology, service innovations can increase the efficiency and quality of the fitting process for HDs, enable remote services outside of professional stores and meet the trends for personalized care and user empowerment.

### Technological and Social Challenges

As service innovations lead to fundamental changes of existing value creation processes between manufacturers, audiologists, and HD users, the development and implementation is faced with numerous technological as well as social challenges. Technological challenges are related to the integrity of the data and the performance of the delivered services. The data that the considered services will use can be highly sensitive, including personal information, location and audio signals. Consequently, the data should be exchanged safely and stored securely. This concern has been one of the main focuses from the conception to the implementation of the technical architecture (cf. section Technical Realization). Concerns for data integrity are driven by increasingly stringent regulations, such as the general data protection regulation (GDPR) acted by the European Union, as well as by end-users being sensitive to these issues. The main challenges concerning the performance of the delivered services come from the difficulty of using advanced signal processing and machine learning algorithms with the limited computational resources which are typically available in HDs. HDs must perform the signal processing in real-time. The incoming signal is processed faster than a latency, called just noticeable difference, so that the HD users do not suffer under issues like reduced lip synchronization. Therefore, keeping the latency induced by signal processing within HDs below a certain level is crucial for the usability. Due to the limited computational resources designing computationally demanding signal processing algorithms for HDs in terms of required operations as well as memory requirements is a challenging task. Algorithms require many iterations for optimal performance, so called convergence. However, algorithms with high memory requirements are not suitable for HDs. Further, the data processed on a HD have a highly sensitive character. Therefore, preserving the privacy of a HD user is important. For these reasons, HDs are not allowed to save any audio material, which can contain clues about the private life of the HD users. Further, it is not allowed to save data which contain features that can be used to reconstruct the acoustic environment. As a result, machine learning algorithms that require large amounts of data to perform optimally have to be incorporated in a HD with caution.

Next to the technological challenges the implementation of digital service innovations can raise challenges on the side of the different user groups. Service innovations that are based on disruptive technological developments have the potential to fundamentally change current value creation processes of audiologists. Disruptive service innovations challenge existing processes, routines and competencies. As competencies shift to technical systems, service innovations can lack acceptance of audiologists. Further, manufacturers in the field of audiology start to enter the service market through digital services. The service developments of manufacturers are often closely related to their product offerings and commercialized as product-service systems (8). Although current digital service offerings of HD manufacturers are distributed and provided through audiologists, the information and data gathered remain on the side of the manufacturers. The outflow of knowledge, experience and user information can further weaken long-term competences of audiologists which leads to resistance and lack of acceptance. However, service innovations will only be implemented successfully if the complementary interaction between manufacturers, audiologists and HD users is developed and managed with respect to the specific requirements of digital service platforms. As such, service innovations require the close interaction between different actors like manufacturers, audiologists and even HD users (9). The collaboration and integration of all actors of the service ecosystem is necessary to ensure the functionality and applicability of previously individual service components on an integrated service platform.

### The “Audio-PSS” Service Platform Approach

**Figure 1:**
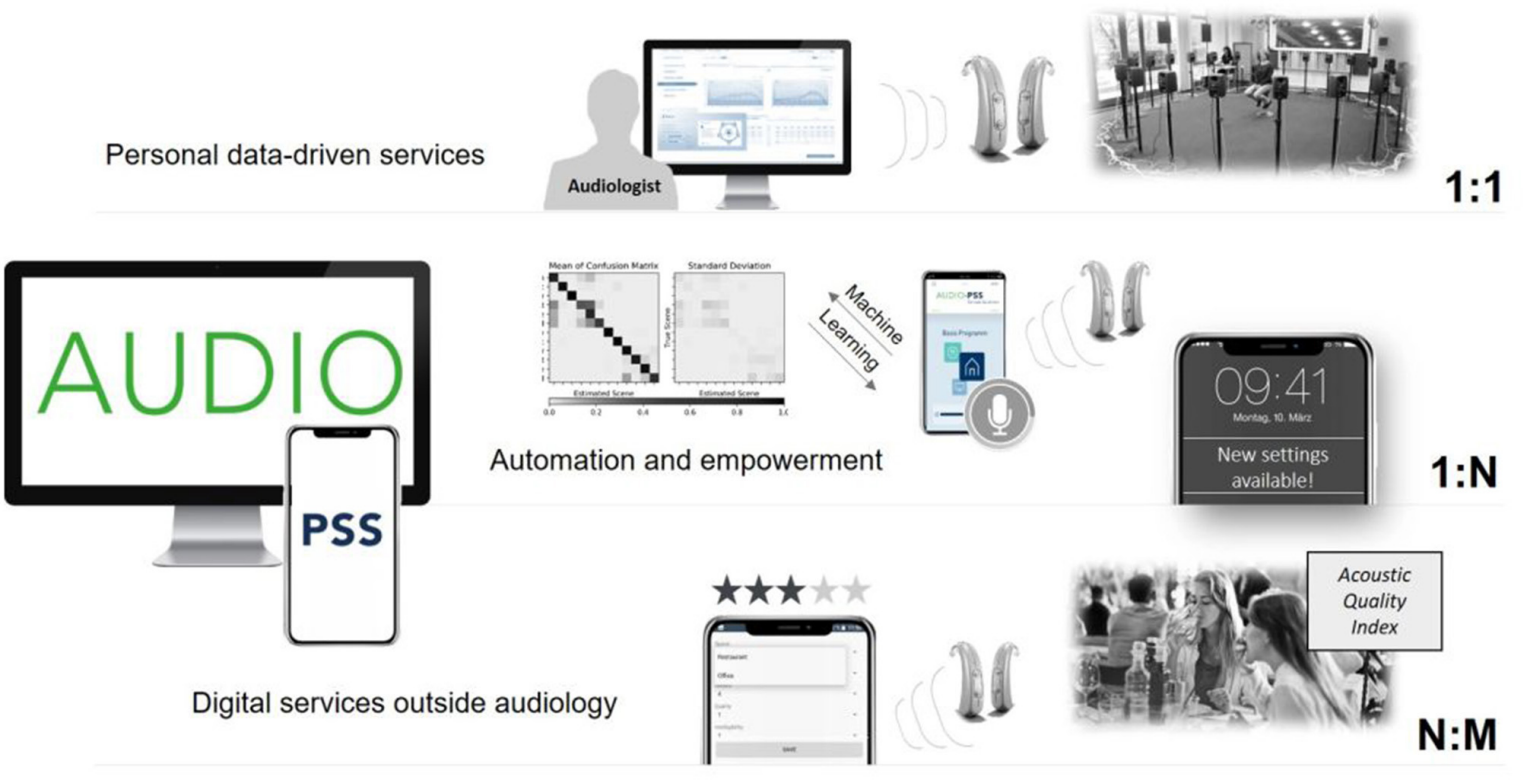
Overview of the Audio-PSS platform approach.

With the joint project *Audio-PSS (development of product service systems in tele-audiology)* we aimed for a participatory development of digital service innovations based on a connected HD which are designed to converge on an integrated service platform. Thereby, we combined advanced technological developments with the important role of human interaction between the audiologist and the HD users. Figure 1 provides an overview of the *Audio-PSS* platform approach that ranges from personal data-driven services (1:1 services) to automation and empowerment (1:N services) and opportunities for new service innovations outside the core field of audiology (N:M services). *1:1 services* are service innovations that aim to improve the relationship between audiologists and HD users, e.g., by allowing an audiologist to continuously monitor HD settings and usage behavior or allow the HD user to contact the audiologist while being in a demanding listening situation for an instantaneous improvement of the settings. The initial fitting, e.g., of HAs, is followed by a familiarization phase in which users collect experiences and impressions of handling HAs in their everyday life. However, the user must memorize these experiences until the next appointment with the audiologist at which they talk about the experiences during the last weeks with the HAs. A common service platform could enable remote online and offline services and could provide documentation of personal experiences for later access by the audiologist. *1:N services* include service innovations that use cloud computing to exchange data, experiences and recommendations of a variety of HD users to automatically fit the HD program through the analysis of aggregated user data. For instance, collective assistance functions can help especially in difficult listening situations by analyzing parameters from the current situation (e.g., location, reverberation, sound pressure level, signal-to-noise ratio) with a machine learning system that was trained offline on aggregated data. *N:M services* are cross-industry innovations that use the data of the connected HD in contexts outside the healthcare domain, e.g., by measuring the acoustic quality of restaurants. For instance, through acoustical monitoring and a simultaneous experience sampling by users; restaurants, cinemas, theaters, educational instructions and other localities as well as public spaces can be recommended with regard to their acoustics, communication experience and well-being, creating an *acoustic quality index*.

In this paper, we provide insights into our project and show the main results of the individual research activities from both the social and technical perspective. From the social perspective, we systematically integrated HD users and audiologists in the innovation process in order to design the service innovations according to their individual needs and preferences. First, we uncovered potentials for service innovations through gaps or specific problems in the current service provision of HD users and audiologists. Further, the user groups contributed to the usability and design of service innovations, e.g., by testing and evaluating different feedback and process scenarios of the remote fitting (RF) concept. With the close integration of audiologists and HD users, we consequently aimed to change the dominating view that digital services substitute personal services. With our audiologist-centered service platform, we show potential pathways how service innovations can create new ways of value creation, improve the quality and interaction within existing service processes and enhance the competencies and opportunities of audiologists in the future service ecosystem. The requirement analysis revealed a great potential for more disruptive service innovations in the context of RF services that audiologists can offer without being physically present. Advanced technological developments like machine learning can increase the efficiency of RF services to the extent that the service can be provided by a service center or by the user alone, independent from personal interaction with an audiologist. As such, there is a great need for a design of the interaction between the HD user, the audiologist, and the technical system that ensures service innovation acceptance.

On the technical side, we show how such service innovations can be technically designed, developed and implemented on one service platform. We designed and implemented a hardware/*s*oftware architecture (10) that allows audiologists to remotely access the HDs of their customers, e.g., for RF, and customers to request support from them. Additionally, this architecture allows to collect a large dataset of audio signals that can be used to improve the performance of HDs. These signals, recorded in a wide range of acoustic scenes, can be used to train advanced acoustic scene classifiers in a remote server and transmit the resulting models from this training to the considered HDs where the recognition can then be achieved in real-time. Though real-time acoustic scene classification in HDs remains a challenging task, the training being done remotely makes it more feasible. As a proof of concept, we developed a machine learning based acoustic scene classifier to automatically recognize acoustic scenes relevant for HD users. For the training of the classifier and the evaluation of the recognition accuracy, we recorded a diversity of acoustic scenes. In order to preserve the privacy of the HD users, we encoded these recordings using sparse features, which cannot be reverse-engineered for reconstructing the original audio. For the training and testing as well as for the real-time evaluation of the acoustic scene classifier, these features were incorporated.

To the best of our knowledge, we are the first who take a participatory innovation development approach to tailor advanced technological developments with specific user-centered preferences within the field of tele-audiology. With our research, we show promising pathways for audiologists to be a major part in the future value chain of service innovations within the HD industry. Beyond that, we show how machine learning can be integrated in an overall architecture and open new opportunities for RF services within audiology. Based on the findings of our experimental evaluation, we demonstrate the benefits of our technical developments regarding their efficacy to enable and improve new ways of RF and the importance of the relationship between the technical system, the HD user and the audiologist.

The remainder of this paper is structured as follows. First, we will present the results of the technology-related requirements for digital service innovations of HD users and audiologists which we collected and analyzed through qualitative (interviews) and quantitative data (surveys). In the subsequent section, we will present the technical infrastructure we realized to meet those requirements. Therefore, we present the system architecture of the connected HD and our core technological developments in the context of RF: acoustic scene classification through machine learning and the overall development and implementation of the RF infrastructure. After the technical realization, we evaluated the technical developments and social aspects with laboratory experiments, which are presented in section Evaluation. We will conclude this article with future directions and mention technical possibilities as well as social implications to increase acceptance among HD users and audiologists in the context of the integrated service platform.

## Requirement Analysis of Hearing Device Users and Audiologists

Innovation through co-creation plays an important role in the healthcare service context. In a collaborative development process, audiologists can be important actors in the development of future innovations as they have broad knowledge and experience in both the product and service world. Additionally, the development of service innovations within tele-audiology also benefits from the integration of HD users as this provides important insights to the user experience during the process of fitting HDs. In the first part of the project, we therefore investigated the requirements of audiologists and HD users to align the technological developments of manufacturers with the needs and preferences of the user groups.

### Exploration of User Preferences and Technological Developments

We applied an explorative empirical approach consisting of qualitative interviews with audiologists and HD users and an analysis of secondary information on recent developments of manufacturers to compare the current developments with the user preferences. First, we identified specific preferences for future service innovations along the opportunities of a connected HD based on 22 semi-structured interviews with HD users and audiologists. The interviews were recorded and transcribed. Subsequently we validated the results on the identified user needs for service innovations during a 2-day workshop with both groups.

**Figure 2:**
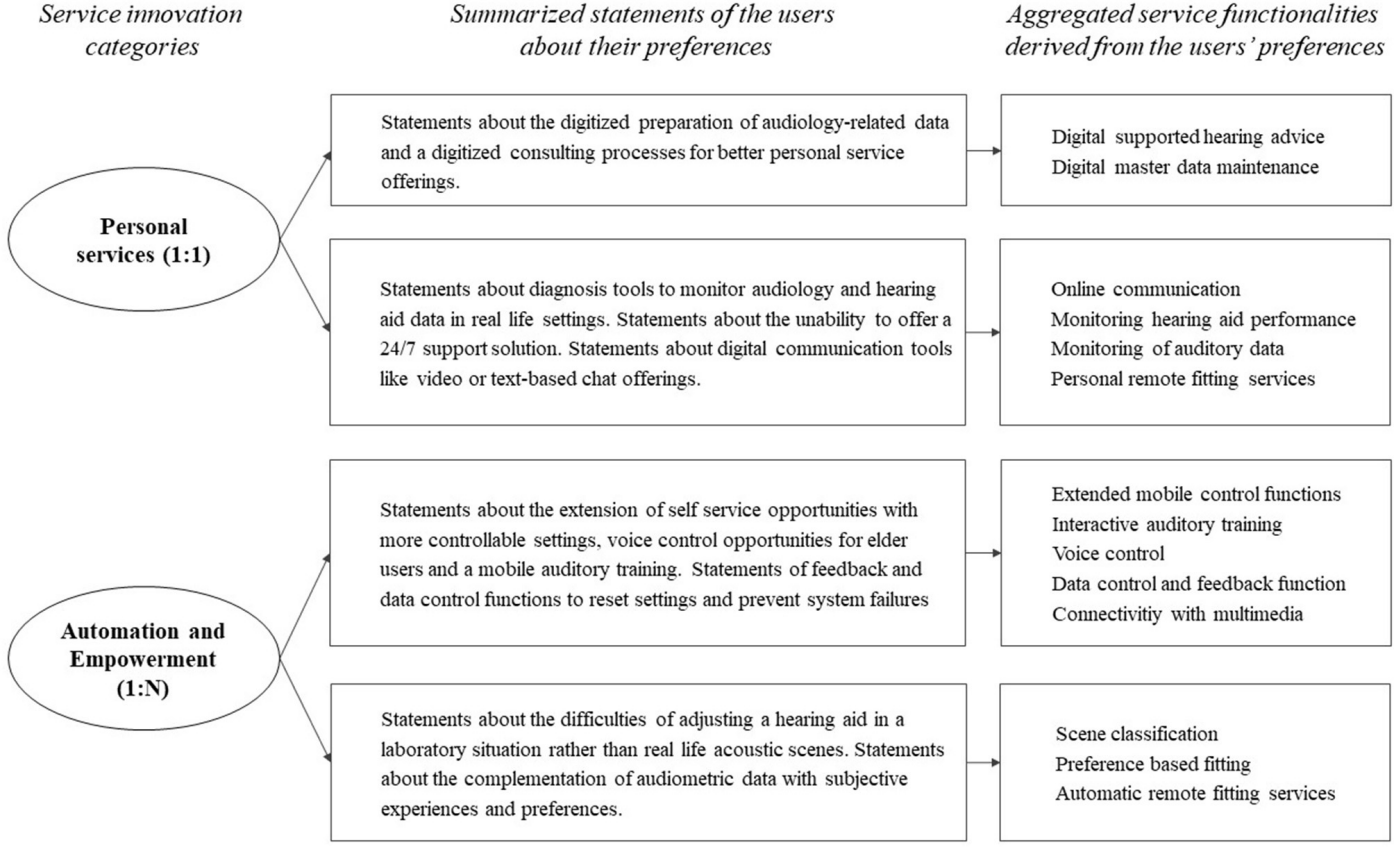
Identification and categorization of user preferences according to their statements in the interview series.

A total set of 85 requirements for future services based on a connected HD has been identified, clustered in 14 different service functionalities. The HD users highlighted requirements for extended mobile control functions, followed by RF services, and for the consideration of individual hearing preferences through scene classification and machine learning. The audiologists highlighted the development and provision of an interactive hearing training, and the development of a scene classification solution in order to monitor the hearing environment and related issues of HD users. Self-services are merely highlighted by the HD users that formulate preferences for services that represent extended mobile control settings: “*I don't really want to adjust anything and certainly not a second device. If these functions were integrated in the smartphone I would say: Great. So many settings on one or two buttons: I'm freaking out.”* Further, a number of HD users and audiologists demand new, audiologist-centered RF solutions: “*Is it possible to bring the audiologist along the various hearing environments I experience? It would be fantastic if the audiologist could adjust my hearing aid remotely, that would be genius.”* Figure 2 shows an overview of the identification and categorization of user preferences. The left side shows the different service categories that have been introduced before. The central column summarizes the coding of the related statements on preferences of the users from the interviews. Finally, the right column categorizes the different user preferences into specific service functionalities.

**Figure 3:**
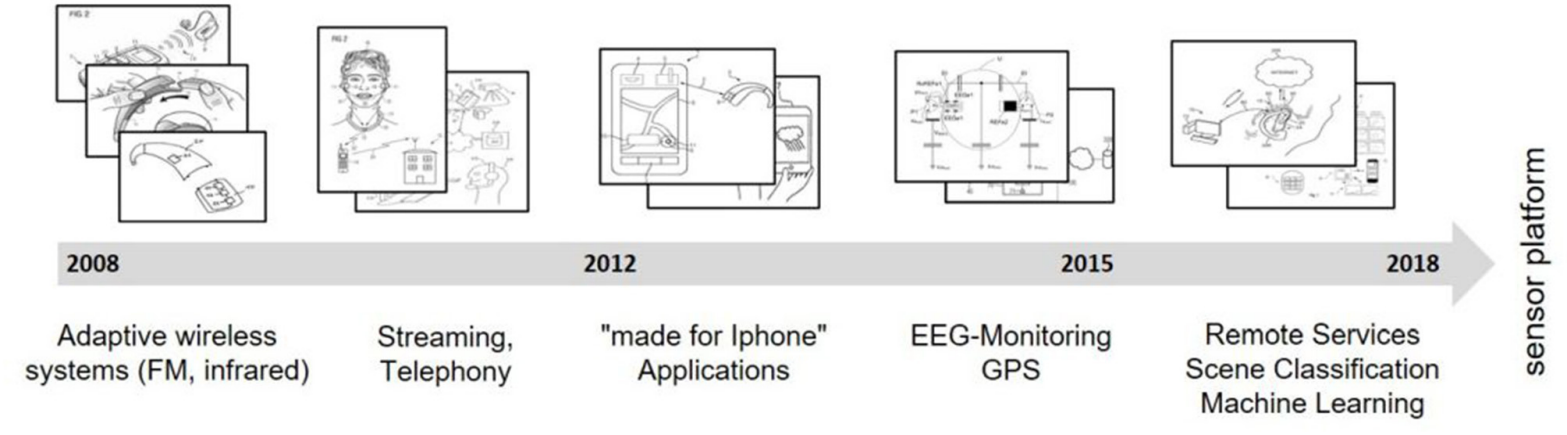
Evolution of the technological trends in the field of audiology.

To identify the current service-related technological developments within the field of tele-audiology, a qualitative patent analysis was conducted (11). The patent analysis was based on the patent database FamPat (Questel). The search period was set from 2008 to 2018. The selected patents included functions about connectivity, use of mobile devices, methods of classification, automation, RF as well as the creation and use of new digital interfaces. In recent years, technological developments focused on the creation of an infrastructure for new digital services from hardware-based rudimentary remote functions in 2008 to machine learning developments in 2018. The patent analysis revealed 21 core technological developments. Within the field of *personal data-driven services* (1:1 services), remote assistance like video chat between audiologists and HD users or chat-based assistance (e.g., WO2013020594) (12) was predominant. More disruptive service developments were reflected by patents on the monitoring of auditory data (e.g., EP3035710) (13) and performance data (e.g., DE102015203288) (14). Further developments enabled intuitive, hands-free interfaces using voice commands (e.g., US2018061411) (15) or physical action based control (e.g., US2014126759) (16) through gestures, eyes, or body movement. With 15 patents, scene classification functions were the most frequently patented technological development in the recent field of automation and empowerment (1:N service, e.g., EP3288293) (17). See Figure 3 for an overview of the developments in tele-audiology since 2008. The figure shows the evolution of technological trends in the field of tele-audiology which were found out with the patent analysis.

Based on the comparison of the user preferences and the service-related technological developments within the field of tele-audiology, our results indicate that manufacturers, HD users and audiologists focus on different service categories. Audiologists articulate more preferences than HD users but focus primarily on personal data-driven services (1:1). Further, the technological complexity often exceeds the capabilities of most users. Currently, many disruptive service innovations that drive new service opportunities originate predominantly from manufacturers.

### User-Centered Design of Different Digital Services

As a result of the explorative pre-study we derived a large set of service innovations expressed by the user groups that are further evaluated within the following two studies. In order to validate the results of the qualitative pre-study we carried out two online surveys with 671 audiologists and 184 HD users. Particularly in the case of 1:N services, different design approaches and beliefs on the future relation between audiologists and HD users were identified, so that we intended to quantitatively determine the specific needs and preferences of audiologists and HD users. Six service innovations were included in both surveys: *Remote fitting* (we distinguished between *time-delayed RF through audiologists* and *instantaneous RF through a service center*), *AI-based assistance function for situation-specific HD optimization, Optimized automatic scene and situation classifier, Hearing Coach, Hearing training*, and *Linkage to social networks*. In the survey of audiologists, we added six service innovations as a result of the patent analysis so that the audiologists evaluated twelve service innovations in the field of tele-audiology in total. The survey focused on the audiologists' intention to offer these services to the customers in the near future. The HD users evaluated eight service innovations and specific design approaches of more disruptive service innovations, e.g., the design of RF services. In this context they had to choose between different specifications of the services, for example between (1) *no remote fitting*, (2) *time-delayed remote fitting by their attending audiologist* and (3) *instantaneous remote fitting by a service center*. Due to the different requirements and preferences expressed in the explorative study, we have adapted the service innovations somewhat to the target group in the quantitative survey.

#### Service Innovation Acceptance of Audiologists

As established healthcare organizations are characterized by strong path dependencies, they typically show a strong dependency upon their mainstream users. While the current service processes typically fit the needs and preferences of existing users, they do not match the increasing demand for digital services and online offerings of emerging target users. As such, service organizations face highly uncertain demands in the future (18) and struggle to balance the ambidexterity between routine and innovation (19). At the same time, service organizations face institutional tensions that hamper the development of future innovation (20). For instance, innovative service organizations face missing reimbursement opportunities for digital services by health insurances, a lack of telemedical regulations, or strict process standards of professional associations. We aimed to integrate the audiologists as providers of service innovations to analyze these challenges further. Therefore, the objective of the study was to find out about audiologists' intention to offer different digital service innovations and their general evaluation of the derived twelve service innovations. The primary research question in this study is: *How does the disruptive potential of service innovations impact innovation acceptance of frontline employees in audiology?*

We surveyed 671 audiologists from different companies and in different positions in Germany. The online survey included questions regarding the acceptance of different service innovations as well as demographic characteristics, digital competence and innovativeness. We used established scales that originate from literature on research of acceptance and innovation management. Within our survey, the audiologists (63% female; mean age = 20–29 years, 56% younger than 30) were employed for 11 years on average (mean = 10.89, SD = 9.41). 14.8% of them were apprentices, 48.6% journeymen/journeywomen, 32.5% master craftsmen/craftswomen and 2.5% others. From a theoretical viewpoint, we distinguished between incremental and disruptive digital service innovations. We classified *six* service innovations as incremental, e.g., *app-based contact, time-delayed remote fitting through audiologist* and *monitoring of hearing aid function*. The remaining six service innovations were classified as disruptive, e.g., *automatic situation classifier, instantaneous remote fitting through service center* and *AI-based assistance function*.

**Figure 4:**
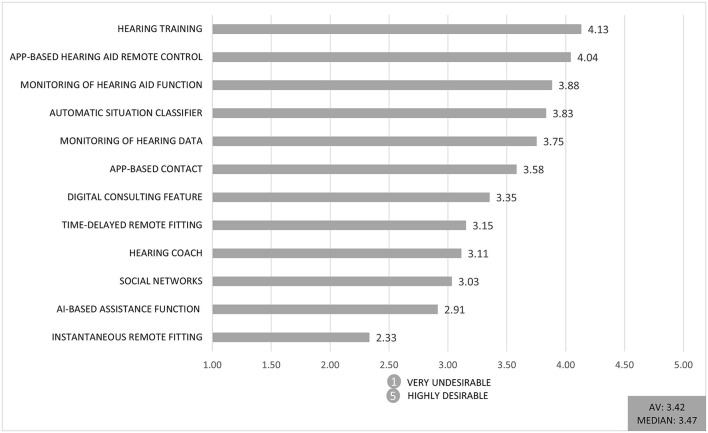
Mean response to the question “*To what extent do you wish to offer the digital service within the next 5 years?”* (1 = very undesirable to 5 = highly desirable; AV = Average).

Only 11% of respondents have a high level of digital competence, and feel confident in using digital tools. This can possibly have a negative impact on the evaluation of disruptive service innovations. But on the other hand 44 % of respondents rate their company as highly innovative. In Figure 4, the twelve digital service innovations are ranked by the intention of audiologists to offer each digital service to their customers within the next 5 years, from *1* = *very undesirable* to *5* = *highly desirable*.

*Hearing training* was rated best among the presented services (4.13 on a 5-point Likert scale). Whereas, *instantaneous remote fitting through a service center* was rated the lowest (2.33 on a 5-point Likert scale). The first service is a complement to the audiologists' work, and therefore can facilitate their daily work, whereas the lowest rated service is a potential threat to their current economic position if the fitting of HDs is taken over by an external service provider. As we have previously classified the services according to their assumed threat potential, this result supports our theoretical perspective that more disruptive service innovations are declined by the audiologists.

**Figure 5:**
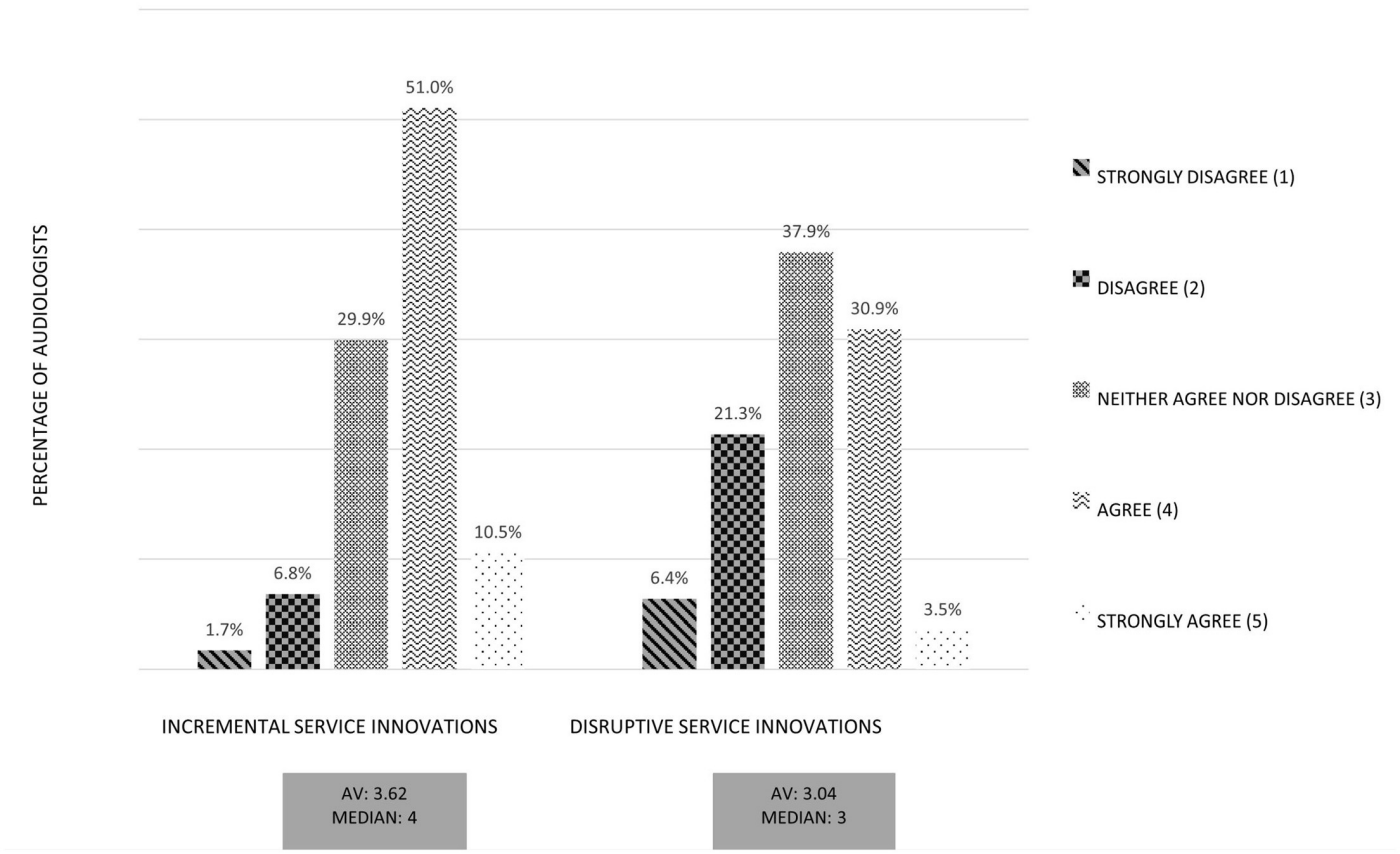
Comparison of incremental service innovations and disruptive service innovations: Aggregated factor Technology Acceptance. Exemplary Item: “*I would like to work with the digital service”*.

As an overarching result, incremental innovations are rated 12% better on average than more disruptive innovations. Figure 5 depicts that more incremental service innovations are rated higher than disruptive service innovations regarding the audiologists' technology acceptance. The acceptance of the service innovations was measured by a multi item scale, containing questions like “*I rate the digital service as useful for my field of work.” “I think my customers would respond positively to the digital service.”* or “*I think that colleagues in my company would advise me to use the digital service.”* The graph shows a summarized factor of the used items for *acceptance of service innovations*. As mentioned above the expectation of change in competence plays a decisive role in acceptance of audiologists.

**Figure 6:**
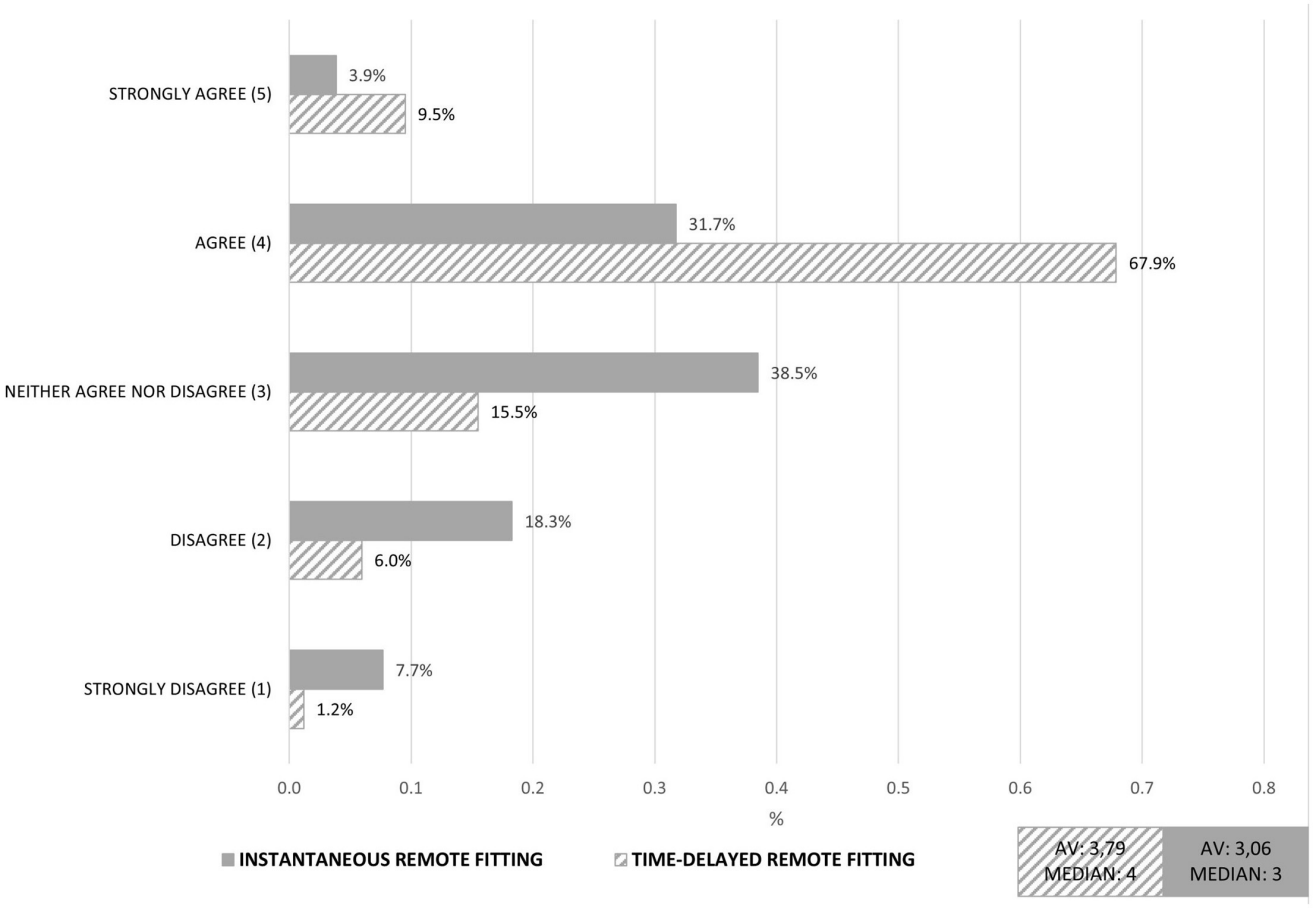
Comparison of instantaneous and time-delayed remote fitting: Aggregated factor Technology Acceptance. Exemplary Item: “*I would like to work with the digital service”*.

Further, we compared the time-delayed RF with instantaneous RF. The RF services differ by (1) a time dimension and (2) a personal dimension. The time-delayed RF was conducted by the personal audiologist whereas the instantaneous RF was conducted by a service center. We hypothesized that for audiologists the fitting by an external service center would be perceived as a threat to their current competences. For this reason we also investigated a contrary RF service in which the audiologist participates strongly. Consequently we defined the time-delayed RF as an incremental service innovation and the instantaneous RF as a disruptive service innovation, from the viewpoint of audiologists. By comparing the two RF services with regard to technology acceptance it showed that the time-delayed RF was evaluated substantially better (see Figure 6). Again, the figure shows a summarized factor of the used items for *acceptance of service innovations*. In particular, the fear of quality losses in the case of RF by a service center had a negative influence on the evaluation of this digital service. Moreover, the service possibly was perceived as a threat for the area of competence by audiologists.

#### Service Innovation Acceptance of Hearing Device Users

In addition to the audiologists, the potential users of these digital services were surveyed. Hearing loss is a sensory disorder that greatly affects the life and social interaction of the affected person. Therefore, many HD users would like more assistance from their HDs in acoustically difficult listening situations, in the handling and adjustment of HDs, as well as in the rehabilitation and acceptance of their hearing impairment. To better understand user preferences, an online survey with HD users was conducted with the goal to identify different service innovations and the participants' willingness to use them in everyday life. The primary research question here was: *Which service innovations and which specifications of service innovations are HD users most likely to use?*

For this purpose, an online survey with 184 HD users (67% female, 85% older than 60 years, median: 70 years) was conducted. All participants were experienced HD users and 78% of them wore their HDs more than 8 h a day. The HD classes that the participants currently use covered basic, mid-range and high-end HDs, which meant that the HDs differed in terms of both technical and comfort features (e.g., noise reduction, situation detection, microphone directionality).

Many modern HDs already offer the possibility to be controlled via smartphone and app. In addition to the technology class of the HDs, the use of smartphones in everyday life is also potentially decisive for the acceptance of this new technology. Eighty percentage of the participants stated that they do not use an app to operate the HD (e.g., program change, volume change) and 14% do not use a smartphone. Regardless of HD control, a total of 54% use their smartphone one to several times a day and 18% one to several times a week, which is an important prerequisite for the use of future digital services. Furthermore, there are technical requirements for HDs to be compatible with digital services (e.g., Bluetooth capability) which are currently not fulfilled by all HD classes. Depending on the HD class and the fixed amount covered by the health insurance, the amount of the private co-payment that HD users have to pay for a technically more advanced HD provision varies. Twenty-eight percentage of participants with public health insurance reported that they paid between 0 and 500 € per ear for their HDs. Thirty-three percentage invested between 500 and 1,500 €, 16% between 1,501 and 2,000 € and 18% more than 2,001 € per ear for a high-class HD. Overall, technology acceptance and personal interest in new technology were rated on average as “partially agree” (3) and “fairly agree” (4) (mean age = 3.43, SD = 0.91), with their own technology competence rated as “less agree” (mean age = 2.03, SD = 0.90). Nevertheless, the participants assess that learning the technique is under their control [M = 3.82 (“fairly true”), SD = 0.90].

- In the questionnaire, the users were introduced to eight different digital service technologies, each in three separate configurations: *Remote fitting* (no remote fitting, time-delayed remote fitting through audiologist, instantaneous remote fitting through service center),
- *AI-based assistance function for situation-specific HD optimization* (disabled assistance function, user-defined adjustment of the HD setting (limited assistance function), automatic situation-dependent optimization of the HD setting),
- *Optimized automatic scene and situation classifier* (disabled automatic scene classifier, user preference-based scene classifier, automatic scene classifier of any acoustic listening situation),
- *Smart home connectivity* [no smart home connectivity, smart home connection with household appliances (e.g., washing machine), smart home connection with household appliances and home technologies (e.g., doorbell)],
- *Hearing Coach* (no hearing coach necessary, hearing coach only virtual in the app, hearing coach virtual in the app and meetings in person),
- *Hearing training* (no hearing training necessary, hearing training only virtual in the app, hearing training virtual in the app and meetings in person),
- *Connection to social networks* (no connection, connection only virtual in the app, connection virtual in the app and meetings in person),
- *Recording of vital parameters* (no recording, recording of vital parameters without exchange with doctor or audiologist, recording of vital parameters with exchange with doctor or audiologist and comparison with other users).

**Figure 7:**
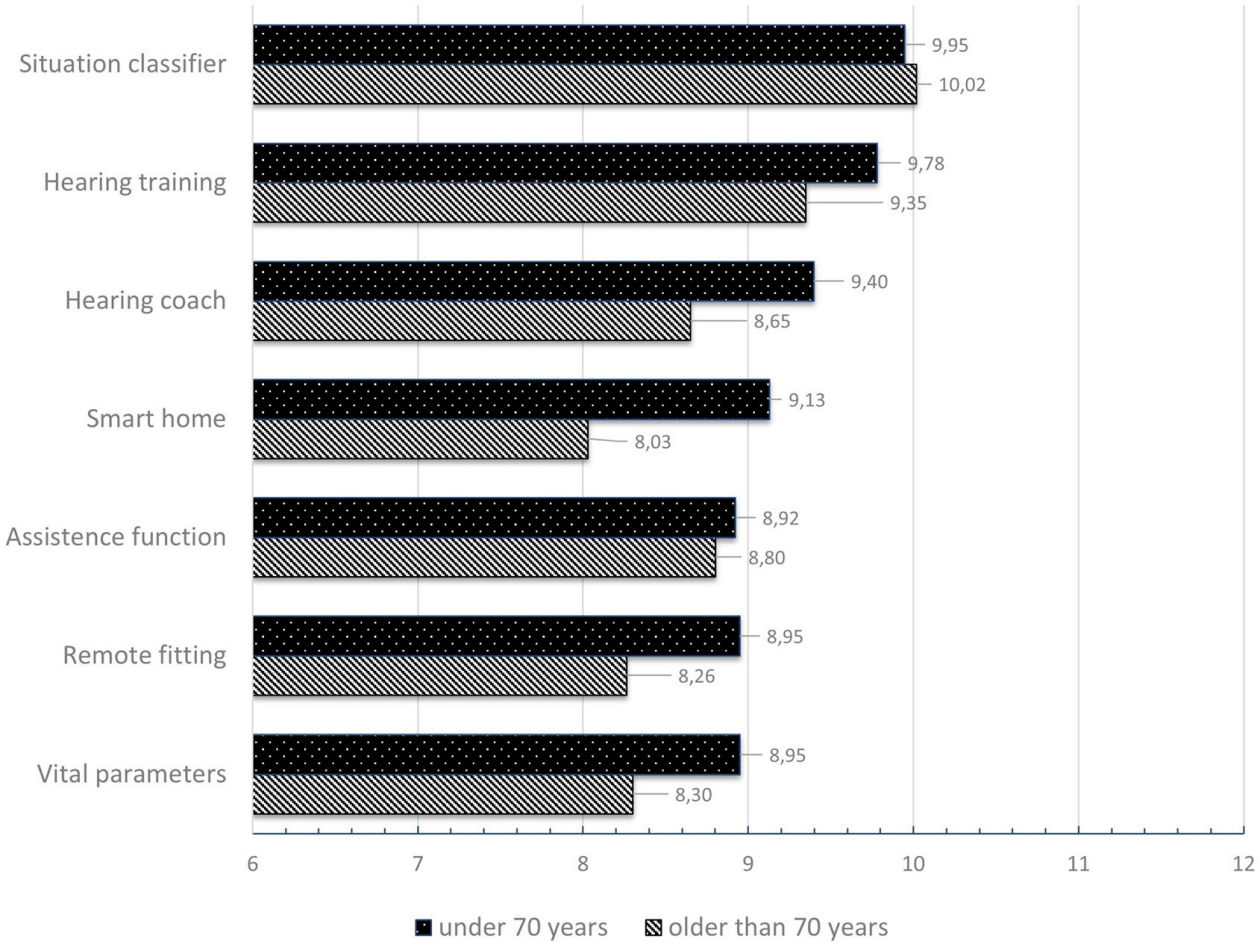
Ranking of the preferred service technologies classified by age.

For the evaluation of the preferred service innovations, the individual scores for the three alternatives were summed (maximum rating = 12, minimum rating = 6). Results were analyzed and ranked for two age groups (above and below 70 years, see Figure 7). Overall, the service *optimized automatic scene and situation classifier* is most desired, followed by *hearing training*. Regarding the *remote fitting services* alone, it can be noted that it is ranked in a split 5th place. However, participants under 70 years are noticeably more open toward the service than those over 70.

The rating of each of the three different alternatives for service innovations: remote *fitting, AI-based assistance function for situation-specific HD optimization*, and o*ptimized automatic scene and situation classifier* can briefly be summarized: The respondents under 70 years of age preferred the function *instantaneous remote fitting through service center*. The older respondents also preferred this type of RF, but also indicated that they actually prefer the face-to-face fitting interview. In terms of technology *AI-based assistance function for situation-specific HD optimization*, both age groups preferred the limited assistance function. They would like to be supported by an AI system, but retain the ability to decide about the HD setting and fitting. In the under-70 group, the *user preference-based scene classifier* specification was clearly preferred over the *automatic scene classifier of any acoustic listening situation*. The participants in the group over 70 years of age, on the other hand, preferred the *automatic classifier*, closely followed by *user preference-based scene classifier*.

### Summary of the Requirement Analysis

The exploration of user preferences and technological developments led to a diverse set of service innovations which we quantitatively analyzed through two surveys with audiologists and HD users. We investigated which service innovations and their specifications are most desired by both groups. It was particularly noticeable that the RF services were evaluated quite differently by HD users and audiologists. Although many HD users preferred the instantaneous RF, the personal component still plays a role, as evidenced by the decision of those over 70 against a RF at all. On the other hand, the audiologists were clearly in favor of time-delayed RF by an audiologist, because they feared a loss of competencies and decreasing quality of customer consultation. For this reason we decided to further clarify the preferences for the different RF specifications and the impact on the acceptance of the adjusted HD fitting. Consequently we followed up the requirement analysis with another evaluation study (cf. section Evaluation). In the social evaluation of RF we gave the HD users three variants to test: RF through audiologist, RF through AI, and a mixed form: RF through AI with approval by audiologists. In technological development, we focused on the mixed form to consider a system that combines both advantages of personal and digital services to meet the preferences of both audiologists and HD users.

## Technical Realization

In this section, the technical realization based on the results of the requirement analysis is presented. We decided for a platform design as HD users and audiologists favor a wide range of functionalities. However, both sides emphasized the importance of a personal relationship, which is why we aimed to offer an integrative platform where all stakeholders are joined. While many functionalities are incremental and can be fed directly into market development, we focused on RF and scene classification, as they require a high level of initial research and development effort. Developing a platform also had the advantage to build and test components for several services independently from each other. The modularity of a platform enables platform providers to adapt or expand their service offerings depending on the market needs as well as technological opportunities. As service platforms offer great interoperability, the HD as well as the collected data can be used for several service solutions provided by the integration of different stakeholders (6). Furthermore, new service innovations can be introduced continuously after the launch of the platform. The overall architecture for the RF approach and the respective subsystems will be described in the following sections.

### System Architecture of the Connected Hearing Device

#### Overview

**Figure 8:**
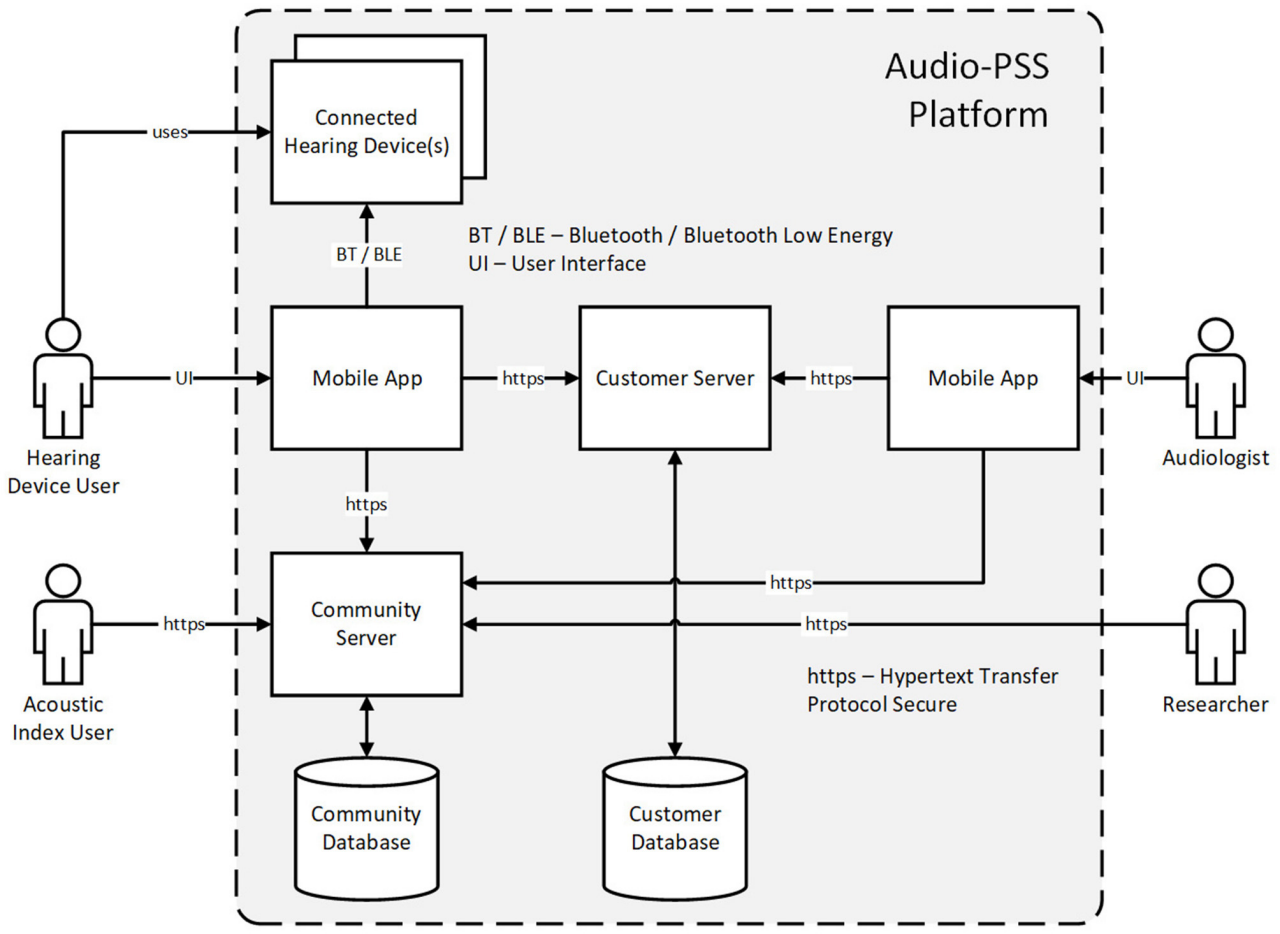
Overview of the platform architecture to support 1:1, 1:N and N:M services.

In order to fulfill the needs expressed by HD users and audiologists during the field study described in section Requirement Analysis of Hearing Device Users and Audiologists, a dedicated hardware/software architecture had to be designed and implemented. We took advantage of the Bluetooth connectivity available in modern HDs to design an architecture that is able to support the one-to-one (1:1), one-to-many (1:N) and cross-industry (N:M) services considered in this paper. Figure 8 depicts an overview of the complete architecture (10). All use cases rely on users accessing one or both of the two used databases, referred to as the customer database and community database.

The customer database is used to store personalized, and hence potentially sensitive, data and can be accessed by HD users and by audiologists i.e., to support 1:1 service innovations. The community database contains anonymized data used to support the 1:N service innovations and cross-industry (N:M) service innovations. Different software clients are provided for each type of users, to visualize and, if relevant, edit the content of the databases. Both, HD users and audiologists, can use mobile applications to access the data. HD users can edit the information about themselves, send messages to audiologists and select new available HD settings. HD users can as well view the acoustic index and make new entries to it. Audiologists can see the information about HD users under their care, answer their messages and change their HD settings during the RF. Acoustic index users can visualize the existing entries using a web browser. Those entries are accessed through a computing cluster that is also used by researchers to improve HD processing, more specifically, to train performant acoustic scenes classifiers. As this paper focuses on the RF application, the next subsections describe the elements of the architecture that support RF, namely the customer server and database as well as the mobile application used by the HD users. The development of acoustic scene classifiers as well as efforts to develop privacy preserving features is described in section Machine Learning for Acoustic Scene Classification.

#### Customer Server and Database

As mentioned in the previous section the customer server is required to store the HD user related data that could be associated with audiological data, such as audiograms or the history of fitting sessions by the audiologist. These data are sensitive with regard to the data protection regulation as they link individuals with their health data. Hence precautions have to be taken to ensure that data is processed in conformity with regulations. In our setup, an SQL server was used. The customer server can be accessed from the app through a secured cloud authentication process (OAuth 2) using encrypted communication (TLS 1.2).

The audiologist uses the customer server to manage the individual history of fittings. Together with the feedback from the user's app (evaluated listening situation as well as sound recordings) the audiologist can create an updated set of HD parameters to improve the HD settings (1:1 service) and send them back to the user. Based on individual proposals and fitting sessions stored and managed from the customer server, the entire content of the customer database can be used to feed the community server, hence supporting 1:N services.

### User Interface for Remote Audiological Services

As a user interface for the patients as well as for the audiologists we provide a mobile smart phone application. It is a core component of the mobile part of the system. It connects the HDs on the one end with servers in the backend and serves as a user interface to gather feedback and display data. By interacting with this app, both stakeholders can perform many tasks, which would usually require a personal meeting, without such a meeting necessary.

**Figure 9:**
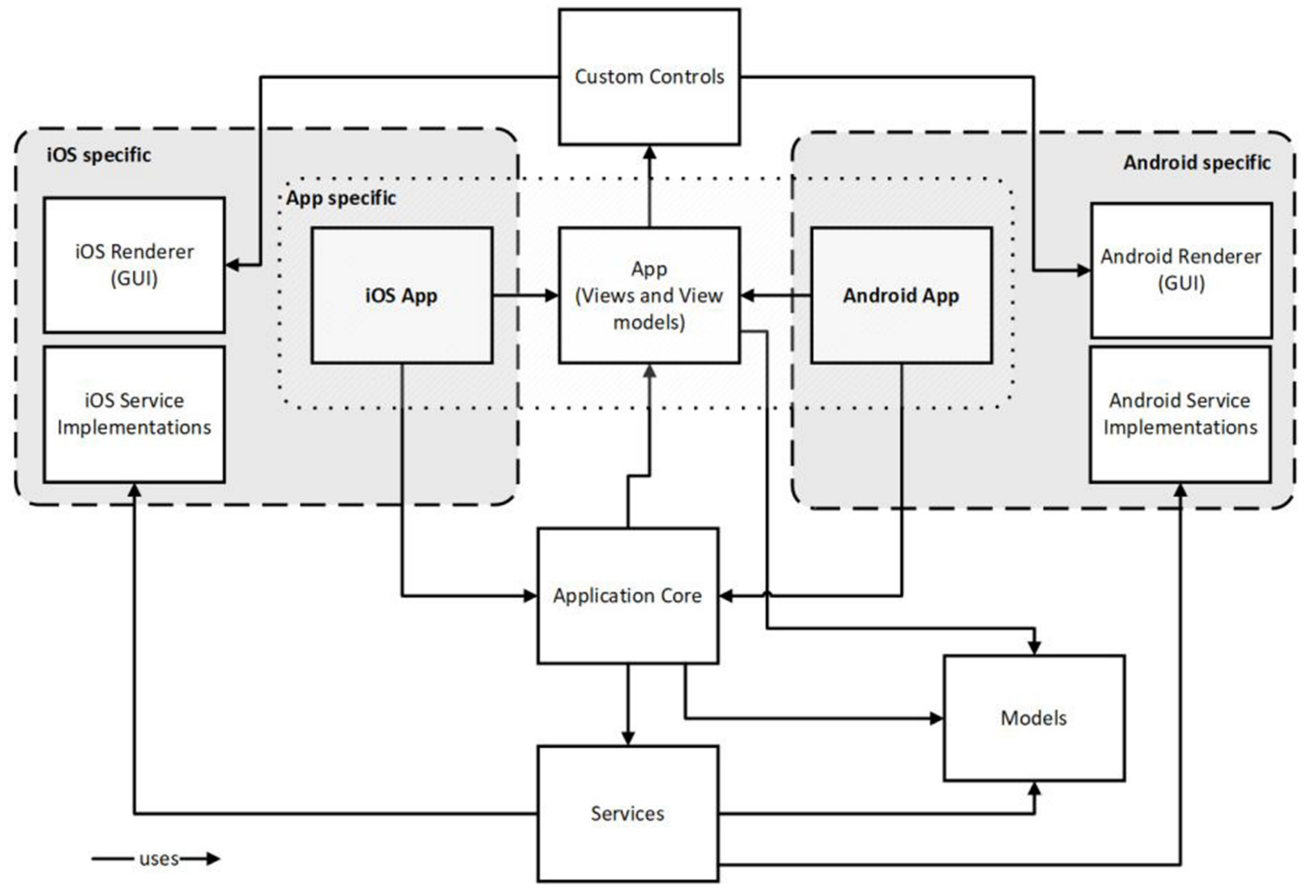
Software architecture of the mobile app.

Having several use cases and functions in mind the Audio-PSS app is designed to be a platform. Designing the app as a platform means on the one hand to create a solution that can be used on different target systems (iOS, Android) without implementing it multiple times. Platform specific concerns like HA drivers and notification systems are dedicated to the particular target system. The other aspect refers to the app as a host for different functions and services. Figure 9 depicts the architecture.

#### Remote Measurement of Hearing Loss

**Figure 10:**
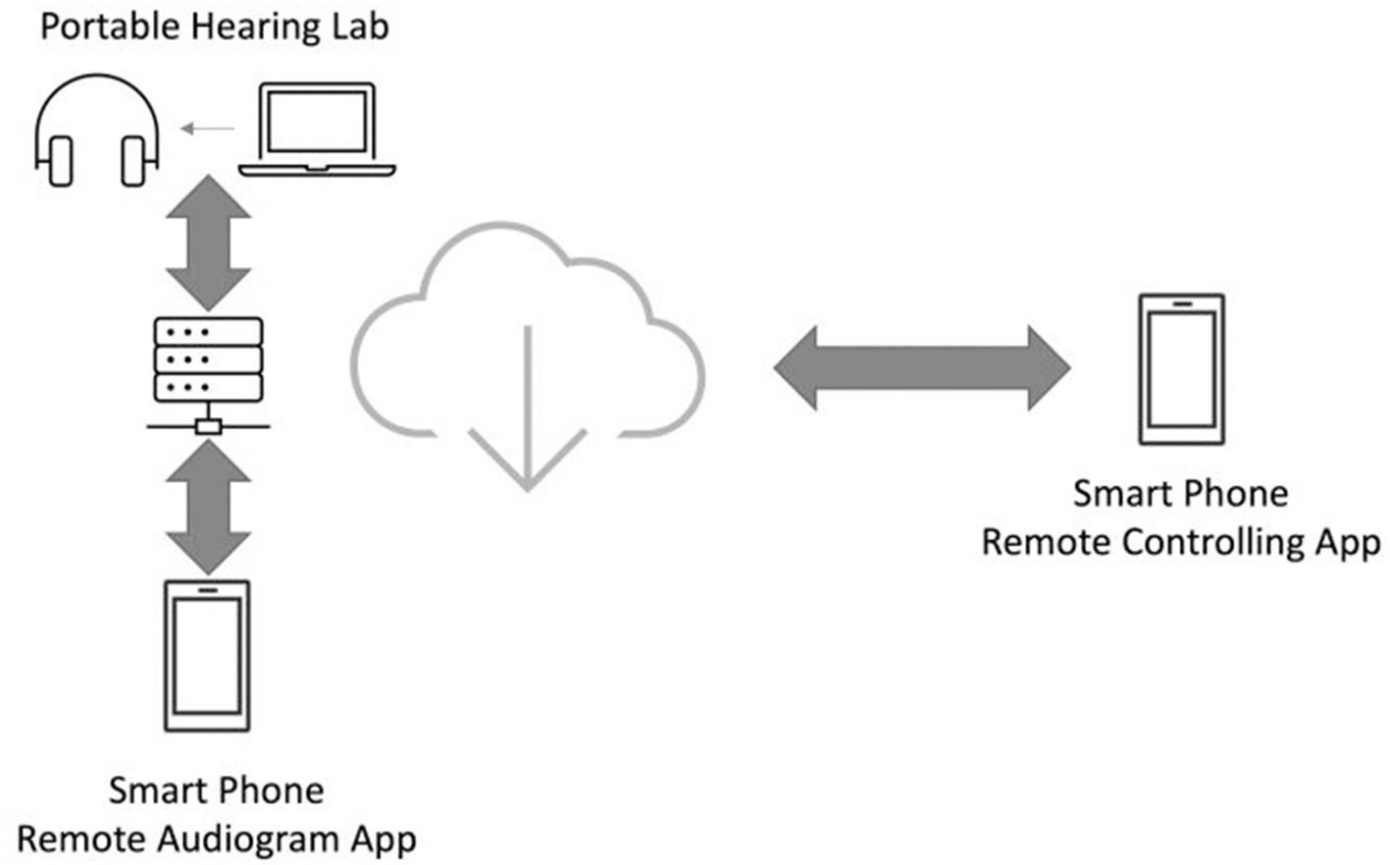
The architecture of the remote audiogram measurement performed using the remote AudioPSS-app.

For the characterization of the hearing loss, typically a pure tone audiogram is measured for both ears. For these measurements calibrated hardware is required. By using the technical infrastructure, we set up a combined solution, which integrates a calibrated hardware/software system with the remote AudioPSS-app. The calibrated hardware is called the portable hearing lab (21). An open source signal processing platform for hearing research called the *open Master Hearing Aid* (22) is running on this platform. This solution provides the audiometer backend of the measurement set up. The architecture is shown in Figure 10.

The remote AudioPSS-app provides two functionalities for measuring the audiogram: by using the first functionality, which is called the remote audiogram app, the patient can perform the audiogram measurement remotely without visiting the audiologist. The second functionality (remote controlling app) is designed for the audiologist to observe the measurement. The audiologist receives the measurement results once the measurement has been completed and can thereby judge the quality of the measurement.

#### Remote Fitting

There are two basic remote fitting scenarios: (a) perform a first fit and (b) execute a follow-up fit. Based on the audiogram data gathered from the remote measurement above the remote audiogram app provides additionally the possibility to perform a first fit using the portable hearing lab device and a selected fitting rule. Hence the patient can get an immediate benefit from the remote measurement.

As for the follow-up fit workflow, a connected user can send his feedback about the current listening situation using categorized symbols and an attached sound snippet record to the backend. This message including the attachments is stored on the customer server. The audiologist, located either in his office or operating from a service center, analyzes the current settings, the listening situation and the user's feedback resulting in a new fitting proposal. The proposal is sent back to the user's smartphone where the user will be notified about it. Then the updated settings can be downloaded, installed and tested. Depending on the testing the new settings can be kept or discarded resulting in another feedback loop with the audiologist.

### Machine Learning for Acoustic Scene Classification

Due to the limited computational resources of HDs the range of acoustic scenes that can be recognized and programmatically treated inside the HD remains limited. In this paper, we introduce a machine learning approach for the recognition of acoustic scenes by incorporating the provided infrastructure for connected HDs to continuously optimize a machine learning model.

In order to train such models, a large training data set is required. In the proposed approach based on the IoT, these sound samples will directly be recorded by the HD users, which raises the question about how to respect the privacy of the HD users. Therefore, we cannot use the sound samples directly to train a machine learning system. State-of-the-art machine learning approaches extract some lower dimensional features (23), which carry the essence of the underlying sound sample. By doing this a simpler machine learning architecture with a smaller number of parameters can be defined. Moreover, we have to make sure that these features cannot be used to reconstruct the underlying sound samples. For this reason, we computed a set of privacy preserving features for training and testing of the machine learning system.

#### Privacy Preserving Audio Features

State-of-the-art scene classification approaches use variants of spectral features in the logarithmic domain, e.g., log-mel spectra or mel frequency cepstral coefficients, for acoustic scene classification (24). However, the underlying audio signals can be reconstructed from these features. For this reason, we decided to use sparse decomposition techniques (25, 26) for encoding the audio signals as sparsely as possible, which account for two important aspects. On the one hand, the characteristics of the underlying acoustic scenes, where these audio signals were recorded, are preserved. On the other hand, an intelligible reconstruction of the underlying audio signals is not possible.

In order to decompose a given audio signal, a Gammatone filterbank (27) is constructed, which is composed of a given number of M Gammatone filters. A Gammatone filter is characterized by its center frequency and bandwidth. It has been shown that the impulse response of the Gammatone filters is similar to the basilar membrane measured in cats (28). Therefore, Gammatone filters are frequently used for modeling the human auditory system.

A sparse signal representation of a given audio signal *x* at time *t* can be approximated as a linear superposition of K Gammatone filters *γ*_*f*_*k*_,*t*_*k*__ called atoms with center frequency *f*_*k*_ and time offset *t*_*k*_, selected from a Gammatone filterbank with M filters is defined as follows:


(1) $$\begin{array}{c} x(t)=\Sigma_{k=1}\;a_{k}\gamma_{f_{k},t_{k}}(t)+\varepsilon (t), \end{array}$$

where *a*_*k*_ is the amplitude of the corresponding Gammatone filter and *ε*(*t*) is the residual. As a decomposition algorithm, we employ the matching pursuit algorithm proposed by Mallat and Zhang (29). The amplitude, center frequency and time offset of the K Gammatone filters $\gamma_{f_{m,\;}t^{*}}$ are defined to be the ones, which maximally correlate with the residual:


(2) $$\begin{array}{c} \left(f_{k},t_{k}\right)=argmax<\varepsilon_{k}(t),\;\gamma_{f_{m},t^{*}}> \end{array}$$

**Figure 11:**
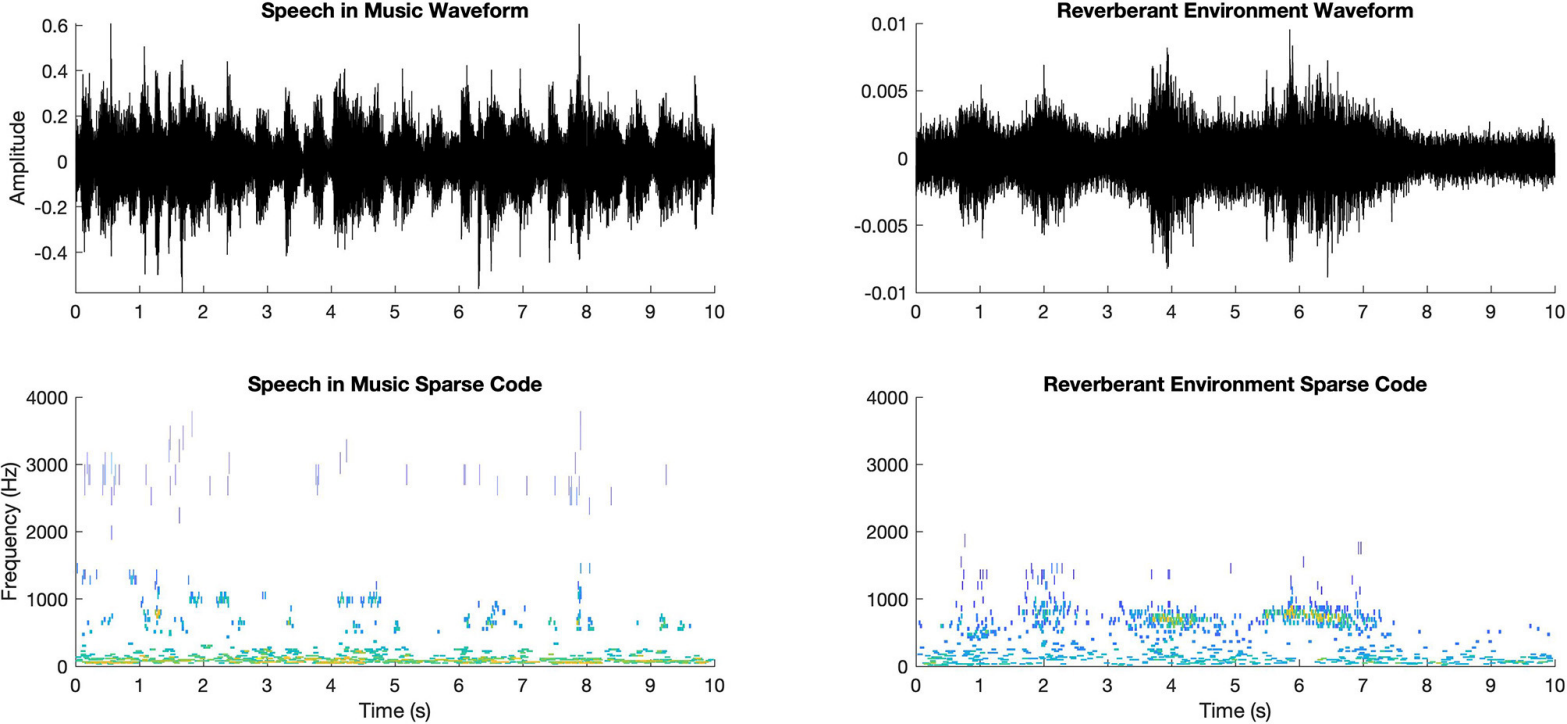
Two sample waveforms (upper row) and the corresponding sparse decompositions (lower row).

The sparse decomposition of a given audio signal in comparison with itself is shown in Figure 11.

For the sparse decomposition of the audio signals, we generated a Gammatone filterbank of M = 64 filters and computed K = 1,024 Gammatone atoms for each audio signal of 10 s.

#### Privacy Preserving Acoustic Scene Classification

In order to recognize a diversity of acoustic scenes automatically, we defined a deep learning architecture using convolutional neural networks (CNNs). In the proposed approach, we aim to train the CNNs on a cloud server in an offline mode and download the pre-trained network to each HD connected to the proposed system.

The defined network architecture consists of four convolutional layers followed by two fully connected layers. The first convolutional layer consists of 32 filters of size 3 × 3. In the following layers the number of filters is doubled with the filter size kept constant. Between each convolutional layer batch normalization and max pooling of size 2 × 2 is applied. After the batch normalization, the rectified linear unit is applied as an activation function. The first fully connected layer consists of 280 neurons. After the first fully connected layer, we apply another rectified linear unit. In order to prevent the network from overfitting, there is a dropout layer with 50% dropout probability between the two fully connected layers. The second fully connected layer is at the same time the output unit consisting of 14 neurons, which is equal to the number of acoustic scenes within the database used for training and testing the system.

## Evaluation

In the following section we present the evaluation of the RF service from a technical as well as from a social viewpoint. In addition we technically evaluated the Acoustic Scene Classification.

### Technical Evaluation

#### Remote Fitting Evaluation (Audiometry App)

In order to evaluate the performance of the RF concept, we performed subjective measurement experiments with 18 normal hearing listeners aged between 20 and 54 years. An audiologist performed three pure tone audiogram measurements with each test subject. Subsequent to these measurements, each test subject performed three audiogram measurements using the remote audiogram app. For both measurement paradigms five frequencies (500, 1,000, 2,000, 4,000, 6,000 Hz) were measured using the audiometry headphones (Sennheiser HDA 200). Both paradigms have a measurement resolution of 5 dBs, which means that in both paradigms the hearing thresholds are increased with 5 dBs steps.

**Figure 12:**
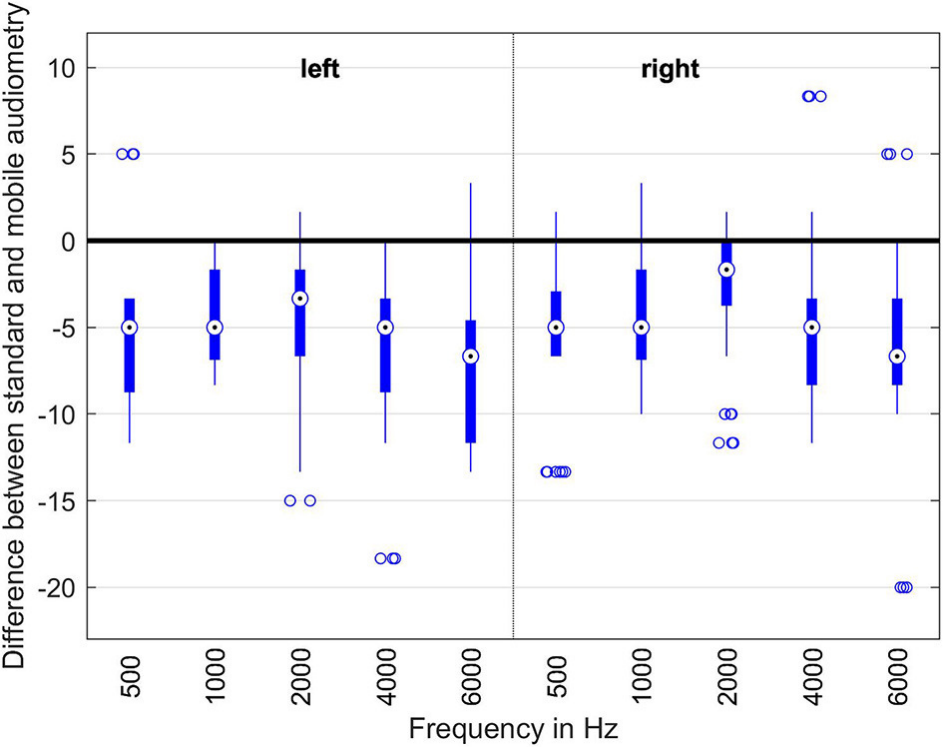
Evaluation of the different audiogram measurement methods. The boxes indicate the measurement points within the 25 and 75% percentiles. The dots within the boxes are the median of all the measurement points for this particular frequency. The thin lines indicate the minimum and maximum measurement points. The dots below and above the thin lines are the outliers.

In Figure 12, the results of the audiogram measurements as a difference between the pure tone audiogram and remote audiogram are shown. The zero line indicates that the measurement results of the pure tone audiogram and the remote audiogram are equal. Negative deviation means that the results obtained with the standard pure tone audiogram indicate higher hearing thresholds, e.g., less hearing loss than those obtained with the remote application.

As one can easily see, the remote audiogram app on average measures hearing thresholds around 5 dB lower than the corresponding pure tone audiogram measurements. The reason for this discrepancy can be explained by the differences between the reaction times in hitting a button or clicking on a smartphone screen. The test-retest reliability in clinical audiogram measurements also typically is in the 5 dB-range. Therefore, the results of the remote audiogram app approximate the pure tone measurements with sufficient accuracy.

#### Evaluation of the Acoustic Scene Classification

In order to train supervised machine learning models, a large training data set is required. While the technical infrastructure for the connected HDs was still in the making, we recorded a binaural data set Hearing Aid Research Data Set (HeAR-DS) (30) to provide a reliable basis to train a supervised machine learning model to perform acoustic scene classification for HDs.

**Figure 13:**
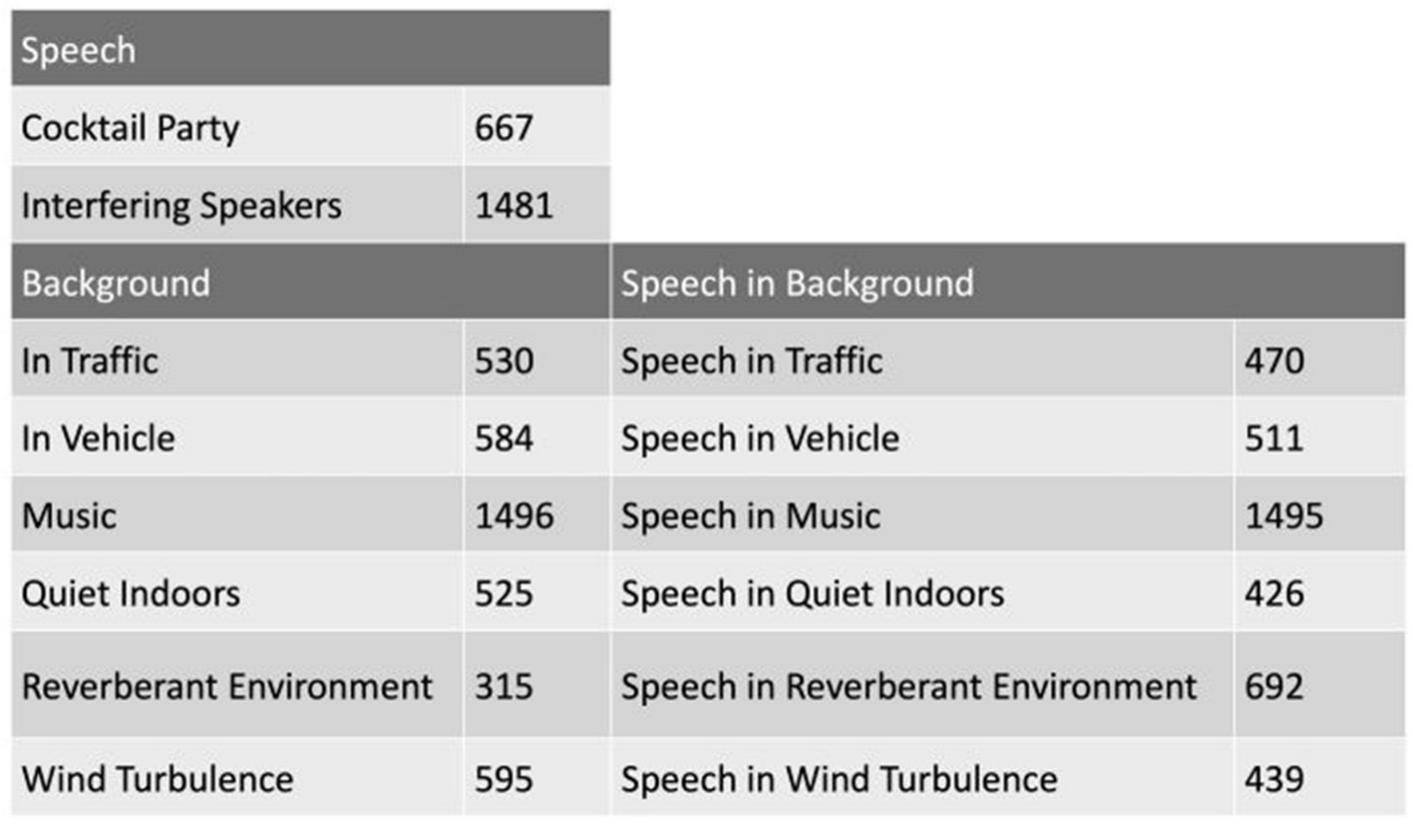
The acoustic scenes of the HeaR-DS research data set is shown. The number next to each acoustic scene indicates the number of samples in the corresponding acoustic scene. Each sample is 10 s. long.

HeAR-DS contains fourteen acoustic scenes as shown in Figure 13 that were defined in close cooperation with audiologists and with the German hearing aid manufacturer and project partner *audifon* to cover acoustic scenes relevant in everyday life of HD users. These acoustic scenes can be categorized in three groups. The *speech* group consists inherently of speech, where the target speaker changes simultaneously and hence is hard to determine. Therefore, *Cocktail party* and *Interfering speakers* belong to that group. The acoustic scenes in the *background* group contain pure background noises (i.e., no significant speech components). Finally, the *speech in background* group consists of acoustic scenes with a target speaker embedded in one of those backgrounds. In order to create the acoustic scenes in this group, background signals were manually mixed with speech signals.

**Figure 14:**
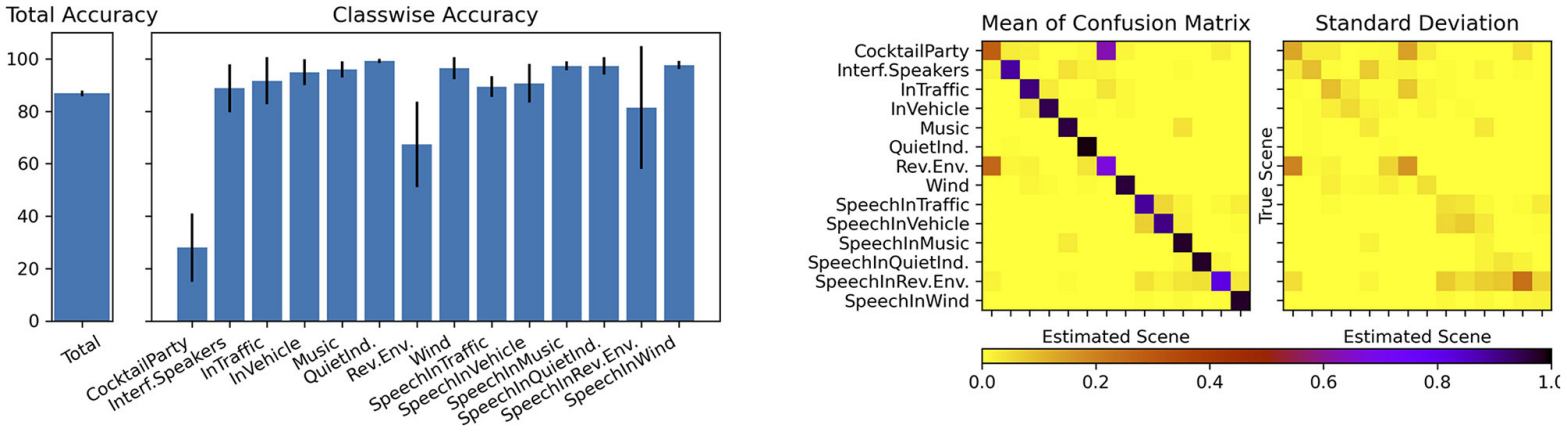
Class wise test accuracies **(left)** and matrix plots showing mean and standard deviation of the confusion matrix **(right)**.

The proposed CNN was randomly initialized, trained and tested 10 times. For the optimization of the weights the Adam optimizer was used. As the size of the training data set is large, we use mini-batches of size 24 samples to train the network. For each training session, a maximum of 250 iterations was performed. An early stopping mechanism was also integrated so that after 10 iterations without any improvement in the loss, the training was terminated. We randomly split the HeAR-DS into training (70%) and test data sets (30%) so that there was no overlap between the sets during each training and test phase. The class wise test accuracies, the confusion matrix and its standard deviation are shown in Figure 14.

The average overall accuracy of all 10 training sessions is 87.26%. All acoustic scenes were successfully classified with high classification accuracies except two acoustic scenes. *Cocktail party* and *reverberant environments* were frequently mixed up with one another. One can easily recognize this phenomenon on the confusion matrix. We hypothesize that the reason for this result is caused by the fact that the sparse codes of these two acoustic scenes are highly similar to one another. There is another slight confusion between the acoustic scenes *speech in traffic* and *speech in vehicle*. As the recordings for the acoustic scene *In Vehicle* were made within different cars in traffic, this confusion is acceptable. The other acoustic scenes were classified with very high accuracies and with very little variance among different training and test sessions.

### Social Evaluation of Remote Fitting

Additionally to the successful evaluation of the technical implementation of RF we also investigated the user preferences and usability regarding the different RF scenarios. The findings of the requirement analysis reveal that the design and interaction between audiologists, HD users and the technical system can be shaped in different ways. The preferences of both audiologists and users suggest a hybrid service process where audiologists, HD users and the technical system interact closely. However, it remains unclear whether such service design exceeds other service interaction processes in terms of user acceptance as well as subjective and even objective hearing quality. Therefore, we experimentally evaluate different RF scenarios with 18 experienced and inexperienced HD users between 59 and 80 years of age (M = 73 years, SD = 5.4 years, six female) with a mild to moderate hearing loss. The objectives of this study were to investigate the preferences of hearing-impaired participants for their HD fitting and whether these preferences influence the perception, measured by objective and subjective methods, when HD fittings are identical for each scenario.

In this study, participants were fitted with audifon HAs lewi R. Two programs were implemented into the HAs: the standard setting program 1 and a “comfort in noise”-setting as program 2. Three different fitting scenarios were investigated in this study: fitting adjustments by (1) an audiologist, (2) fitting by the AI system, and (3) fitting by both the audiologist and AI system. To ensure comparability of the measurement results, the modification of the fitting in all three scenarios was realized by switching from preset program 1 to program 2. For the experiment, three different acoustically complex listening situations were set up in the laboratory using eight circularly arranged loudspeakers, in which the subject had the task to repeat the sentences presented and to fill in a questionnaire afterwards. For the fitting adjustment, the probands were asked to either verbally describe their hearing problems in the listening situation (scenario 1) or enter them into the app for the AI system (scenarios 2 and 3). Subsequently, the setting in the HAs was changed by switching programs unnoticed by the participant and the test was repeated.

The objective speech intelligibility tests performed in each scenario turned out very similarly for all three fitting scenarios (*mean word scoring in %:* scenario 1: 69%, scenario 2: 70%, scenario 3: 72%). The subjective questionnaires also show that the study participants did not notice any perceptible differences in, among other things, speech understanding, listening effort, or sound based on the different fitting scenarios. Furthermore, participants were asked to rate “*Which type of remote fitting would you prefer?”* (ranking from 1 to 3).

**Figure 15:**
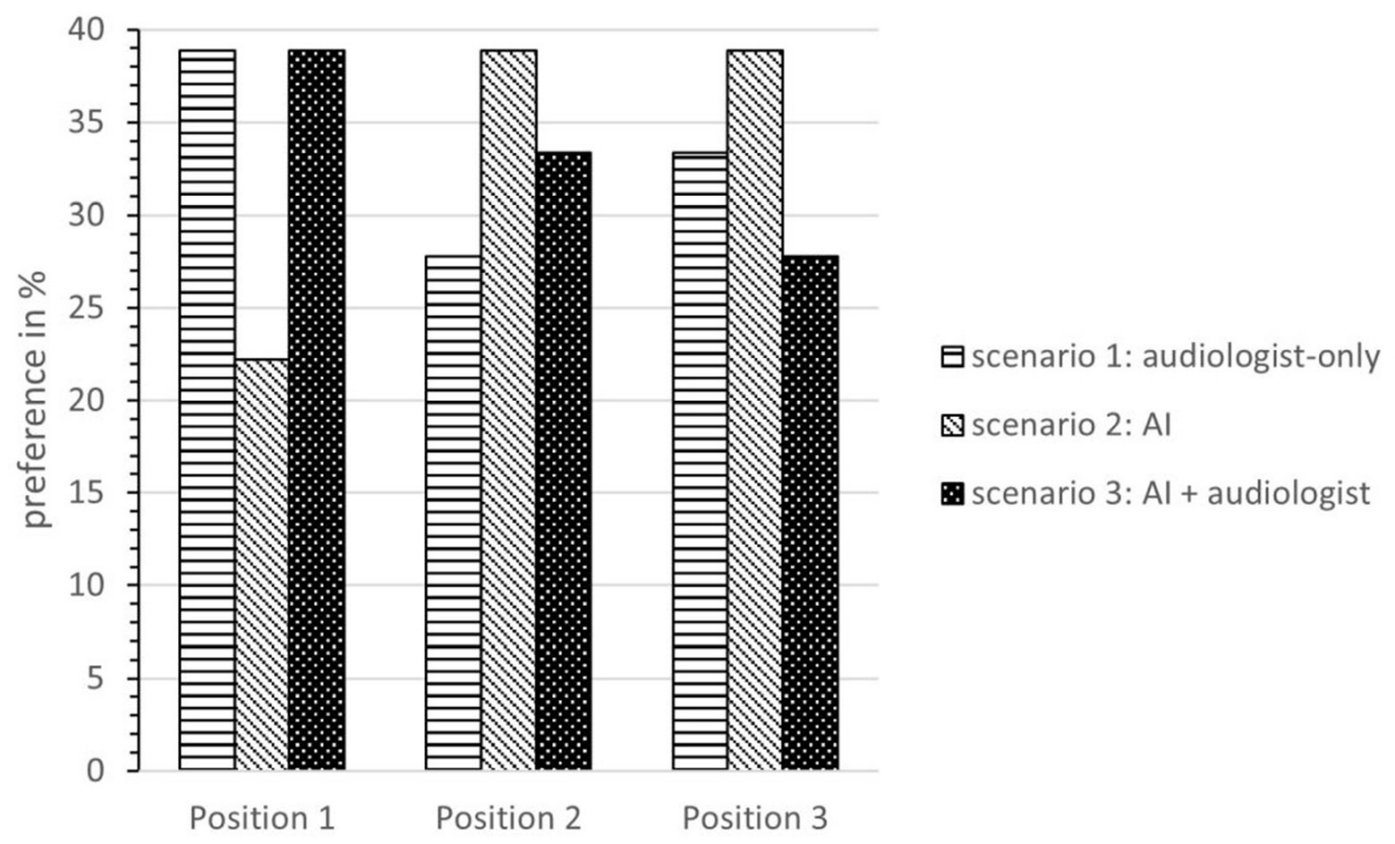
Preference ranking of the three fitting scenarios.

The results show that 38.9% of the participants prefer the mixed option (scenario 3: AI + audiologist) for adjusting their HAs, and 38.9% state that the audiologist-based fitting is their favorite. Only 22.3% preferred the scenario with an AI-only automated fitting. Overall, 72.2% of participants ranked the mixed option first or second, whereas only 66.7% said this was the case for the audiologist-only option. Figure 15 depicts the preference ranking of the three fitting scenarios. In conclusion, the HD users did not notice any difference in the objective parameters but have a clear preference for a technical solution in which the audiologist is still actively involved. This is seemingly in contrast to the data from the requirements analysis, where the HD users preferred the instantaneous RF through a service center (see section Service Innovation Acceptance of Hearing Device Users). However, those over 70 years old rated the classical consulting on site (no RF) as the best alternative. And in the explorative interviews, HD users stated that the personal component should not be replaced. Since the objective evaluation of the RF scenarios did not differ, the tendency toward a technical solution, supported by an audiologist, was strengthened.

## Conclusion and Future Outlook

In this paper, we presented our concept of an integrated service platform for digital service innovations in audiology. At the beginning of the project, we identified the requirements of HD users and of audiologists on different digital services. Based on the user needs we focused on the technical infrastructure needed to meet those requirements. In this context we presented the system architecture of the connected HD and the acoustic scene classification through machine learning as well as the overall development and implementation of the RF infrastructure. To conclude the user-centered developments, we evaluated the acoustic scene classification from a technical standpoint and the RF solution from both technical and social standpoints. With our interdisciplinary study, we developed digital service innovations based on connected HDs which converge on an integrated service platform. With our platform approach we focused on advanced 1:1 and 1:N services as RF and acoustic scene classification. For the participatory innovation process we combined advanced technology developments with the important role of human interaction between audiologists and HD users.

We found out that the preferences of HD users, audiologists and manufacturers differ with regard to potential digital services. The HD users highlighted preferences for self-services, followed by RF services, and for services on the 1:N domain like the consideration of individual hearing preferences through scene classification and machine learning, whereas the audiologists approved particularly an interactive auditory training, and the development of an acoustic scene classification solution. An audiologist-centered RF solution was mentioned by both user groups. Based on the comparison of the user preferences and the patent analysis of service-related technological developments, we found that more disruptive service innovations mainly originate from manufacturers. With the subsequent user surveys the trends of the explorative analysis were supported: The audiologists preferred more personal data-driven services, whereas the HD users preferred self-services and services on the 1:N domain. For RF services it was particularly noticeable that they were rated quite differently by the user groups. Audiologists clearly preferred time-delayed RF by an audiologist, whereas many HD users preferred the instantaneous RF. Nevertheless, the personal component still plays a role, as evidenced by the decision of those over 70 against RF.

We developed a hardware/software architecture that can support all service dimensions (1:1, 1:N and N:M) underlying the project. Relying on state-of-the-art database development and communication protocols, we ensure that this architecture is both safe and scalable. Indeed, the architecture design allows to easily increase the number of users, both HD users and audiologists, as well as the connections between them, e.g., allowing each audiologist to support a larger number of HD users. Consequently, though we tested this technical solution only on a limited number of participants, it could easily be applied to a much larger user group. One of the main applications of this architecture is RF, whose proof of concept has been evaluated in the form of a remote audiogram measurement. Measuring the pure tone audiogram remotely was a key step for realizing the RF approach. The evaluation studies have shown that our proposed remote audiogram app is able to measure the hearing thresholds within the normal test-retest reliability of audiogram measurements. It also enables a first fit of the HDs remotely and is therefore a convenient tool for the RF process.

For the social evaluation we experimentally evaluated three different fitting scenarios: fitting adjustments (1) by an audiologist, (2) by the AI system, and (3) by both the audiologist and AI system. The objective speech intelligibility tests performed in each scenario turned out very similarly for all three fitting scenarios. In the subjective evaluation of the different RF scenarios, the mixed scenario scored the best, which shows that HD users are open toward a technical solution but still prefer the integration of a personal component.

As our results show, the development of a service platform for digital services in healthcare requires interdisciplinary collaboration. With our project team we combined expert knowledge from audiology, information technology, and service research. With the combination of social and engineering science, we made valuable implications for the further developments of digital services in the field of tele-audiology. Moreover, with multiple stakeholder integration, we showed how a service platform can connect advanced technological developments to users and audiologists. For manufacturers, we highlighted the necessity of integrating audiologists and HD users in the development of future service processes and offered a system architecture that integrates all relevant stakeholders on the same service platform. For audiologists, we raise awareness for the topic of digitalization in audiology and show that audiologists should take an active part in the development of digital service innovations. The early alignment and participation in service innovation processes ensures the sovereignty and importance of audiologists in future service processes. But most of all, the platform approach opens up new alternative ways of value creation. The re-programmability and homogeneity of digital innovations can be used to create service innovations beyond the core field of audiology. For instance, cross-industry innovations on the basis of the hearing data can be used for an acoustic index by evaluating the acoustic quality of restaurants.

The connected HDs paradigm technically allows to perform many measurements, which typically take place on site remotely without the physical presence of the HD user. These kinds of measurements offer a large flexibility to the HD users as well as to the audiologists. The results of the remote audiogram study showed that HD users can measure audiograms remotely and perform the first fit for their HDs based on the measurement results themselves. The possibility to monitor and accompany the measurement by an audiologist remotely reduces the risk and gives the HD user more confidence. Currently, the remote audiogram app depicts only one possible usage of the technology of connected HDs concerning the 1:1 services scenario. However, many other measurements can be handled using the same infrastructure in the future.

Acoustic scene classification indicates a possible application of the 1:N services scenario using the connected HA technology. While increasing the speech intelligibility, preserving the privacy of the HD users has the highest priority in the HA signal processing. The sparse features encode the essence of an audio signal, while preventing a reconstruction of the audio. Hence, these features are very well-suited for tasks, which require collection of data for post-processing from individual HD users. The results (87%) of the conducted acoustic scene classification experiments showed that the proposed sparse features with deep learning architectures turned out to perform in the same range as the state-of-the-art log-mel features (85%) (30), for this task. A slim CNN architecture provides sufficient accuracy for recognizing an acoustic scene. The recognition happens every 10 s, which is frequent enough for a smooth performance of a HA, because the acoustic scene typically does not change more frequently.

The hardware/software architecture should be further evaluated in a wider field study taking advantage of its scalability. This would allow us to confirm the robustness of the developed architecture as well as to confirm or infirm the conclusion made in this paper. Additionally, such a study would allow us to prove the feasibility of the acoustic index and to measure the interest of the end users for this service. Moreover, a usability study could allow us to improve the user interface of the mobile application to make it easier to use. Exemplary usability evaluation methods are a “cognitive walkthrough” where experts evaluate the ease of use as well as a “usability testing” of the demonstrator by potential end users. The importance of user friendliness of such an application on the acceptance of the provided services indeed remains to be quantified.

To further quantify the results of the first RF evaluation we currently survey HD users toward their intention to use different RF options, with regard to the status quo during the Covid-19 pandemic but also in perspective after the pandemic. Future research can also focus on the use and development of sensor data, to further optimize connected digital services beyond the scope of audiology, e.g., wearable devices like fitness tracker. In this context future research can extend the core field of hearing improvement to other use cases in the context of telemedicine, which has become increasingly important since the Covid-19 pandemic.

## Data Availability Statement

The datasets presented in this study can be found in online repositories. The names of the repository/repositories and accession number(s) can be found below: https://www.hoertech.de/en/research/open-tools-for-science/417-hoertech/englisch-ht/projects-ht/open-mha/770-hear-ds.html.

## Ethics Statement

The studies involving human participants were reviewed and approved by Research Ethics Committee of the Carl von Ossietzky University of Oldenburg for non-medical research projects. The patients/participants provided their written informed consent to participate in this study.

## Author Contributions

ML, CG, and MKr collected the data and performed the data analysis for the requirement analysis. BC and UM implemented the architecture used for the project. BC focused on the backend while UM focused on the development of the mobile application. KA collected the data and performed the data analysis for the technical implementation and evaluation of Remote Fitting and Acoustic Scene Classification. MKr collected the data and performed the data analysis for the social evaluation. ML coordinated the manuscript draft and the service sections. KA coordinated the technical sections. ML, CG, KA, MKr, BC, and UM wrote the main parts of the article. MT and MKi revised and completed the article. CS designed the methodological approach, supervised the project and revised the final manuscript. All authors contributed to the article and approved the submitted version.

## Funding

This research was part of the Audio-PSS project (www.audio-pss.de) funded by the German Federal Ministry of Education and Research (BMBF) within the Program Innovations for Tomorrow's Production, Services, and Work and managed by the Project Management Agency Karlsruhe (PTKA). The grant numbers are: 02K16C200, 02K16C201, 02K16C202, 02K16C203, and 02K16C204.

## Author Disclaimer

The authors are responsible for the contents of this publication.

## Conflict of Interest

MKi was employed by the company KIND GmbH & Co. KG. UM and MT were employed by the company audifon GmbH & Co. KG. The remaining authors declare that the research was conducted in the absence of any commercial or financial relationships that could be construed as a potential conflict of interest.

## Publisher's Note

All claims expressed in this article are solely those of the authors and do not necessarily represent those of their affiliated organizations, or those of the publisher, the editors and the reviewers. Any product that may be evaluated in this article, or claim that may be made by its manufacturer, is not guaranteed or endorsed by the publisher.
